# Definite Causes of Caries

**Published:** 1882-04

**Authors:** J. S. Cassidy


### DEFINITE CAUSES OF DENTAL CARIES.

BY J. S. CASSIDY, D.D.S.

(Read before the Mississippi Dental Society, March 2, 1882.)

Mr. President and Gentlemen:—When our committee selected this first subject on the programme, it was our intention, if possible, to obtain the views of Dr. Watt, but, inasmuch as the doctor felt the uncertainty as to whether he would be able to attend the meeting, my colleagues of the committee suggested that I might be able to offer some thoughts on the subject.

The decay of teeth, according to not infrequent allusions of Holy Writ, of the earlier writers among the sages of the Vedas, and to Shakespeare's pen picture of the ancient Roman mob, has probably co-existed, with other forms of human disease from the beginning of recorded time. It can only be a matter of possible conjecture as to what extent the human family in past ages suffered with this now general affliction but I most assuredly presume to disagree with Dr. Kingsley, of New York, in the opinion that the teeth of each succeeding generation are departing more and more from nature's standard. There is undoubtedly a decided increase in æsthetic culture in our branch of the human family, but the tendency to dental caries is not more marked among the highly refined classes, except in the minds of the exclusive operator, who rarely has an opportunity to investigate the comparative extent of that infirmity among the humbler, rugged classes of our people. This is an assertion with which perhaps many of you cannot agree; I am sure, however, that every dentist whose general clientelle includes all classes and colors will support me in this opinion.

Mere mental or physicial refinement do not predispose to structural deficiency of the teeth, nor to the production of an immediate cause of their decay, any more than to cancer, dyspepsia, or a thousand other ills to which man is liable.

There are, however, systemic or constitutional conditions, either inherited or acquired, and held in abeyance more or less by vitality, which permit or perhaps induce the development of the special agent to act as the direct cause of the disease.

It is now universally conceded that acids are the direct cause of dental caries, capable of overcoming both the chemism of the molecules and the watchful care of vitality in the part attacked. To Dr. Geo. Watt, of our association, is due the honor of introducing the acid doctrine of dental caries, and giving us definite knowledge as to which particular acid causes a given variety of decay. Although a firm believer in this doctrine, proven to my mind by proper reasoning and processes of analogy, a lengthy intermitted series of experiments in the mouth have not apparently resulted so as to aid me in arriving at such a conclusion. I have yet to find the first instance of a well marked acid reaction in the mouth, and in conditions too, most favorable for testing in expectancy of its presence.

This fact suggested the question "is it necessary that the oral fluid should appear acid in order to produce our typical disease? and if so, to what extent?"

Believing that certain experiments out of the mouth could render a satisfying answer, I prepared three teeth, by covering their entire surface with gutta-percha, then cutting round smooth apertures through the latter, thus exposing different points on the enamel surface; at some of these points the enamel was removed by instruments, laying bare portions of the dentine also suspending the teeth so prepared in wide-mouthed vials, each containing 15,00 c c ( $\frac{1}{2}$ ) of distilled water at a temperature of 37° c (98° F.) Dr. Watt's Sampsons were selected, and added as follows: o. 06 c c. (1 minum) of strong nitric acid to the water in one of the vials; to the second and third vials, was added the same quantity (o, 06 c c.) of strong sulphuric and hydrochloric acids respectively, and then all three of the vials were placed in a favorable position near a furnace so as to retain as near as possible an equable temperature of 37° c.

At the end of seven days, the teeth were dried and carefully examined, and no appreciable change on either the exposed enamel or dentine could be detected, although each liquid exhibited a very palpable acid reaction to test paper.

The quality of acid was then doubled in each case (o, 12, c c of acid, to 15,00 c c of water), and a tooth having no protecting covering, also placed in each vial. After seven days trial the several teeth were again examined, but no visible change had been produced.

Another increase in the acids of 6 f. cts. making each solution o, 18 c c of acid to 15,00 c c, of water was tried for one week. The enamel of the unprotected teeth was found somewhat roughened, more by the hydrochloric acid than by the nitric or sulphuric acids, and more by the nitric than by the sulphuric acid.

The unprotected points of enamel on the teeth covered by gutta-percha were not apparently affected, but the exposed dentine in these latter teeth was perceptibly softened, though to no great extent, and in the order named in the other class.

The time employed in these simple experiments may have been too brief; the immediate approach of this meeting having prevented the report of a more extended research. However, the only question in doubt is, would the weaker solution, if given longer time, be able to accomplish any solvent action on these teeth?

In all probability they would not, although their per cent. ($\frac{3}{8}$ and $\frac{3}{4}$ of 1 ) nearly reached the strength of lactic acid solution required by Magitot (1 ); but his one per cent. of lactic acid involved a measure of one liter of water (two quarts) while in the experiments just detailed only 15,00 c c (one-half fluid ounce) were employed in each case, thus approximating the average quantity of fluid present in the mouth, which to my mind is an important item.

The fact of our inability to find a general well-marked acid reaction in the mouth need not lead us to suppose that acids are not therefore, the exciting causes of dental caries. Processes of fermentation and putrefaction may be controlled or entirely stopped in any one of their several stages by an untoward change in the nature or condition of even one simple or compound substance in the mass, or by the non-admission of an external though essential element, or the admission of a substance capable of disturbing the plans so nicely arranged by the mutual affinities of the acting bodies. But if either process be permitted to run its natural course, it will certainly terminate in the acid belonging to its peculiar type, at which stage fermentation and putrefaction, properly so called, are ended.

And so, also, in the mouth, if non-interference were the rule here, there would be no difficulty in finding, in abundance, the typical acids of the decomposing substances. Happily, however, interference, in many ways, is the rule.

The intended limits of this paper will not allow respectable mention of these various anti-acid influences save one, and that one, in my opinion, the most important of all in this connection, namely: undisturbed normal vitality. The vital force governing the oral cavity is just the same in kind as that which watches over the health of the cavities of the nares, ears, or other parts or organs, its ability to govern properly, as it desires, here or there, differing only in degree.

If each kind of caries is directly caused by definite chemical decomposition of the part, it is only by the inability of vitality to resist the development of the given acid; if the acid be developed in contact with a tooth, a molecule of the substance of the latter, even of the best enamel, is withdrawn at the point where the molecule of acid has its birth, and instantaneous union of these opposite molecules takes place in proportion to the equivalent value of the acid molecules so developed.

If the acid molecule has its birth beyond the area of attraction between it and the osseous molecule, it finds a plentiful supply of basic radicles in the saliva, mucus, or remains of food, and thus it disappears.

The hard dense enamel on a living tooth is impervious to the enemy only in so far as to present no points of surface in which decaying matter can long remain The sound healthy teeth of either the old or youthful, that we so much admire, have not, of themselves, successfully resisted the attacks of acids, simply because there have been no nascent acids there to attack them. We give credit to their evidently good structure, and contrast them with the sickly ones already more or less destroyed; while, in reality, as seems to me, they remain undisturbed merely because vital force has thus far been able to properly govern their neighborhood, preventing the arrival of the acid stage of fermentation, although in all other respects the local conditions were most favorable for its coming.

Nature prevents disease by arresting or changing the process that develops the existing cause, and in proportion to its variable power in this direction is manifested the varying degrees of predisposition to disease.

The dentist of the future may discover, in each case, the lacking qualities, and so be able to supply vitality with the physical aid necessary to enable it to successfully resist whatever special tendency there may be to dental caries.

